# Expression of Transketolase like gene 1 (*TKTL1*) predicts disease-free survival in patients with locally advanced rectal cancer receiving neoadjuvant chemoradiotherapy

**DOI:** 10.1186/1471-2407-11-363

**Published:** 2011-08-19

**Authors:** Juliana Schwaab, Karoline Horisberger, Philipp Ströbel, Beatrice Bohn, Deniz Gencer, Georg Kähler, Peter Kienle, Stefan Post, Frederik Wenz, Wolf-Karsten Hofmann, Ralf-Dieter Hofheinz, Philipp Erben

**Affiliations:** 1 III. Medizinische Klinik, Universitätsmedizin Mannheim, Theodor-Kutzer-Ufer 1-3, 68167 Mannheim, Germany; 2 Chirurgische Klinik, Universitätsmedizin Mannheim, Theodor-Kutzer-Ufer 1-3, 68167 Mannheim, Germany; 3 Institut für Pathologie, Universitätsmedizin Mannheim, Theodor-Kutzer-Ufer 1-3, 68167 Mannheim, Germany; 4 Klinik für Strahlentherapie und Radioonkologie, Universitätsmedizin Mannheim, Theodor-Kutzer-Ufer 1-3, 68167 Mannheim, Germany

**Keywords:** hypoxia, radiochemotherapy, rectal cancer, *TKTL1*, *VEGFR-1/2*

## Abstract

**Background:**

For patients with locally advanced rectal cancer (LARC) neoadjuvant chemoradiotherapy is recommended as standard therapy. So far, no predictive or prognostic molecular factors for patients undergoing multimodal treatment are established. Increased angiogenesis and altered tumour metabolism as adaption to hypoxic conditions in cancers play an important role in tumour progression and metastasis. Enhanced expression of Vascular-endothelial-growth-factor-receptor *(VEGF-R*) and Transketolase-like-1 (*TKTL1*) are related to hypoxic conditions in tumours. In search for potential prognostic molecular markers we investigated the expression of *VEGFR-1*, *VEGFR-2 *and *TKTL1 *in patients with LARC treated with neoadjuvant chemoradiotherapy and cetuximab.

**Methods:**

Tumour and corresponding normal tissue from pre-therapeutic biopsies of 33 patients (m: 23, f: 10; median age: 61 years) with LARC treated in phase-I and II trials with neoadjuvant chemoradiotherapy (cetuximab, irinotecan, capecitabine in combination with radiotherapy) were analysed by quantitative PCR.

**Results:**

Significantly higher expression of *VEGFR-1/2 *was found in tumour tissue in pre-treatment biopsies as well as in resected specimen after neoadjuvant chemoradiotherapy compared to corresponding normal tissue. High *TKTL1 *expression significantly correlated with disease free survival. None of the markers had influence on early response parameters such as tumour regression grading. There was no correlation of gene expression between the investigated markers.

**Conclusion:**

High *TKTL-1 *expression correlates with poor prognosis in terms of 3 year disease-free survival in patients with LARC treated with intensified neoadjuvant chemoradiotherapy and may therefore serve as a molecular prognostic marker which should be further evaluated in randomised clinical trials.

## Background

Neoadjuvant chemoradiotherapy has become standard treatment for locally advanced rectal cancer due to improved local tumour control. Distant metastases are currently the predominant cause for treatment failure [1]. Therefore, the search for prognostic and predictive markers has been widely promoted in the last few years [2,3]. To date, no validated prognostic or predictive molecular marker in the setting of locally advanced rectal cancer has been established.

Angiogenesis as a central process in progression of solid tumours is a well-established aspect of cancer biology [4]. Inhibition of involved tyrosine kinase receptors such as the epidermal growth factor (EGFR) and the vascular endothelial growth factor receptor (VEGFR) or its ligand VEGF is effective in several tumour types [5,6]. *VEGFR-2 *is believed to be the major mediator of angiogenesis in human tumours, whereas *VEGFR-1 *is said to play its primary role during embryogenesis and regulates apoptosis and tumour growth in malignancies [7]. Several studies have outlined a trend towards more aggressive tumour growth in terms of distant metastasis in patients with VEGF-overexpressing rectal cancer undergoing neoadjuvant treatment [8]. However, expression data of the different VEGF subtypes and their receptors in colorectal cancer still remain controversial [9-11] and their prognostic impact on patients treated with neoadjuvant cetuximab-based chemoradiotherapy has not yet been evaluated.

Many cancers show a strongly enhanced glycolytic metabolism of carbohydrates even in the presence of oxygen ("aerobic glycolysis"), a phenomenon firstly described by Nobel laureate Otto Warburg ("Warburg effect") [12]. The detection of the Transketolase-like-1 (TKTL1) protein and its role in the pentose phosphate pathway (PPP) first described a link between enhanced glycolysis and cancer [13]. Increased *TKTL1 *expression on mRNA and protein level correlates with poor patient outcome and metastasis in many solid tumours [14-18]. Specific inhibition of *TKTL1 *mRNA has been shown to inhibit cancer cell proliferation in functional studies [14,17].

In the present study, we aimed to analyze the potential prognostic and predictive influence of *VEGFR-1/2 *and *TKTL1 *expression on early response parameters such as pathological tumour regression grading (TRG) and tumour downstaging and on 3-year disease-free survival in patients with LARC undergoing cetuximab-based chemoradiotherapy within clinical trials.

## Methods

### Patients and Treatment

The present analysis comprises patients with histologically confirmed, locally advanced non-metastatic rectal adenocarcinoma (endorectal ultrasound stage cT3-4, any N or cT2, N+ distal rectum). All patients participated in clinical trials of intensified neoadjuvant chemoradiotherapy including weekly irinotecan (40 - 50 mg/m^2^) and cetuximab (initial dose of 400 mg/m^2 ^then 250 mg/m^2^), and daily capecitabine (400 - 500 mg/m^2 ^b.i.d.) in combination with pelvic radiotherapy (45 Gy + 5.4 Gy) as previously described [19,20]. Follow up of patients was carried out according to the German study group for gastrointestinal Oncology [21]. Patient characteristics are listed in Table 1.

**Table 1:** Patient and tumour characteristics in 33 patients treated with cetuximab based chemoradiotherapy

	Patients (n)	%
**Patients included**	33	100

**Median age (range)**	61 (33 - 76)	

**Gender**		
**Male**	23	70
**Female**	10	30

Performance Status (ECOG*)		
**0**	23	70
**1**	9	27
**2**	1	3

**Tumour marker**		
**CEA, median (range)**	2,4 μg/l (0.5 - 50,3)	
**CA 19-9, median (range)**	10 kU/l (1 - 298)	

**Clinical T-staging (TRUS**^**§§**^**)**		
**cT2**	6	18
**cT3**	24	73
**cT4**	3	9

**Clinical N-staging (TRUS**^**§§**^**)**		
**cN negative**	10	30,3
**cN positive**	23	69,7

**TRG**^**§ **^**(JSCCR**^**┼┼**^**)**		
**0**	1	3
**1a**	6	18
**1b**	4	12
**2**	19	58
**3**	3	9

§§: Transrectal ultrasound; §: Tumour regression grade; ^┼┼ ^= Japanese Society for Cancer of the Colon and Rectum

Patients provided written informed consent for the participation in the clinical study as well as for the investigation of biopsy material. The clinical study protocol was reviewed and approved by the local institutional review board and the study was performed according to the Declaration of Helsinki.

### Pathological assessment and definition of tumour response

Surgery was performed 4 - 6 weeks after the completion of chemoradiotherapy. The pathological routine work-up was described earlier [22].

Two classification systems were applied to describe response to chemoradiotherapy. The grade of histopathological regression has first been described using the Japanese Society for Cancer of the Colon and Rectum (JSCCR) grading system [23]. Tumours were classified as good-responders when assigned to tumour regression grades 2 or 3 (TRG 2 or TRG 3), and as bad-responders at regression grades 0 or 1 (TRG 0 or TRG 1).

Moreover, the histopathological downstaging after completion of preoperative chemoradiotherapy was used as surrogate parameter of tumour response, as previously described by Valentini et al [24]. A T-level downstaging of at least one T-level was considered to be a sign of response.

### Tissue samples, Real-time quantitative polymerase chain reaction

Tumour material was obtained during rectoscopy before the initiation of chemoradiotherapy and during surgery of the primary tumour. Tumour biopsies (n = 33) and matching healthy mucosa (n = 33) were stored in RNAlater (Quiagen, Hilden, Germany) in liquid nitrogen, and stored at -80°C until further extraction. RNA extraction and cDNA synthesis was performed according to standard protocols [25]. Total RNA was extracted after homogenisation of 15-30 mg tissue with the Ultra Turrax Tube Drive (Ika, Staufen, Germany) using TRIzol™ reagent (Invitrogen, Karlsruhe, Germany) according to the manufacturer's instructions. RNA was reversely transcribed using random hexamer priming and MMLV reverse transcriptase (Invitrogen).

Expression analysis of *VEGFR-1*, *VEGFR-2 *and *TKTL1 *was performed using the LightCycler instrument 1.5 (Roche Diagnostics, Mannheim, Germany). For sequences of amplification primers and hybridisation probes used see additional file 1. Each 20 μl reaction mix contained 4 μl LightCycler Faststart DNA Masterplus Hyb Probes Master Mix (Roche Diagnostics), 2 μl cDNA template or plasmid dilution, 0.5 μM forward primer and 0.5 μM reverse primer, 0.25 μM anchor probe and 0.25 μM sensor probe (TIB Molbiol, Berlin, Germany). Cycler conditions were the following: 10 min denaturation at 95°C, 50 cycles of 10 sec at 60°C (annealing *VEGFR-1, -2*, and *TKTL1*) and 26 sec at 72°C (elongation). A 5 log series of plasmid dilutions was amplified within the PCR runs for quantification of *VEGFR-1, -2 *and *TKTL1*. The LightCycler software prepares standard curves using linear regression analysis of the plasmid dilutions and calculates copy numbers of the unknown sample [26]. Values below the lowest standard dilution for *TKTL1 *(4 copies) and *VEFGR-1/2 *(40 copies) were assigned as negative. Beta-Glucuronidase (*GUS*) mRNA was quantified as internal control as previously described [27].

### Cloning of Quantification standards

Cloning and transformation of the PCR products of *TKTL1*, *VEGFR-1 *and *VEGFR-2 *amplified from cell lines (SW480, K562 obtained from DSMZ, Braunschweig, Germany) was performed according to the manufacturer's instructions using the PCR2.1 TOPO vector (Invitrogen). Amplification reactions were undertaken for 32 cycles at 60°C annealing temperature. Cloning and transformation into Escherichia coli TOPO10F' was performed according to the manufacturer's instructions (Invitrogen). Plasmid DNA containing the desired construct was isolated using the Plasmid Mini Kit (Qiagen). Insertion sequences were confirmed by direct sequencing. The resulting plasmid was linearized by Not1 digestion (Roche Diagnostics). Dilutions of the linearized plasmid were prepared in 10 mM Tris-HCl (pH 8.0) and 1 mM ethylenediaminetetraacetic acid containing 20 mg/mL tRNA (Roche Diagnostics).

ß-Glucuronidase (*GUS*) mRNA transcripts were measured as an internal control using a standard plasmid (pME-2) containing *BCR-ABL*, *ABL*, and *GUS *sequences [27]

In order to minimize dilution error of different plasmids containing target and housekeeping gene a common plasmid containing *TKTL1 *and *GUS *was constructed for further use using a previously published method [27].

### *KRAS, PTEN *and *Survivin *analysis

*KRAS *mutation analysis was performed for all samples by direct sequencing from DNA of microdissected tumor tissue samples as described [28]. *PTEN *status was determined by immunohistochemistry (IHC) using the *PTEN *antibody as described [28] (1:400, Cascade Bioscience, Winchester, MA, USA). All 33 patients were screened for *PTEN *mutations, 30 of which were evaluable. Three samples could not be taken into the analysis due to poor sample quality. Survivin expression analysis was performed on cDNA level of 30 patients from the studygroup using qPCR.

### Statistical analysis

Differences in expression levels were compared using two-tailed Mann Whitney test. Differences in regression rates concerning the investigated tissue markers were evaluated by means of a two sided Fisher's Exact test. Disease-free survival was defined as the time between the start of chemoradiotherapy and tumour relapse (local failure and/or distant metastases) or death due to non-tumour related causes using the Kaplan-Meier method. Cut off level of median gene expression was used to divide low and high expressing groups. A p-value of less than 0.05 was considered statistically significant.

## Results

### Patients, tumour characteristics and expression levels in tumour tissue

Patient and tumour characteristics are depicted in Table 1. Tumour tissue of a total of 33 patients (n = 23 male, n = 10 female, median age 61, range 33 - 76) was analysed. *TKTL1 *expression was determined in 33 patients, while *VEGRFR-2 *expression was analysed in 32 and *VEGFR-1 *expression in 26 patients only due to scantness of tumour tissue.

**Figure 1:**
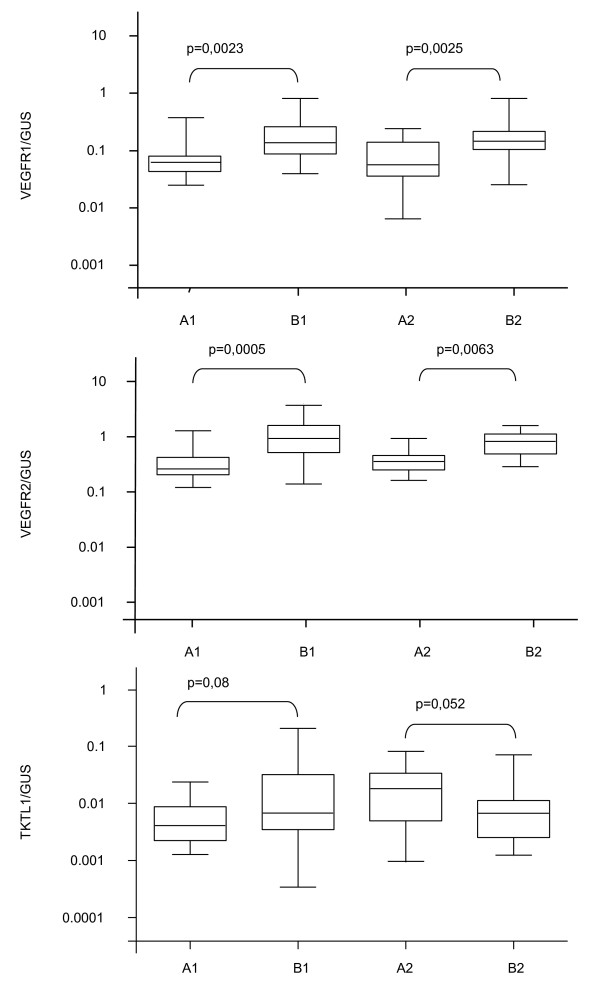
**Comparison of normalised median expression levels of *VEGFR-1*, *VEGFR-2 *and *TKTL1 *in tumour and healthy mucosa**. Comparison of normalised median expression levels of *VEGFR-1 (VEGFR-1/GUS)*, *VEGFR-2 (VEGFR-2/GUS) *and *TKTL1 (TKTL1/GUS) *in tumour and healthy mucosa before (A1, B1) and after neoadjuvant treatment (A2; B2) (Box and whiskers plot, 10-90^th ^percentile). A1: normal mucosa before neoadjuvant chemoradiotherapy; A2: normal mucosa after neoadjuvant chemoradiotherapy; B1 tumour tissue before neoadjuvant chemoradiotherapy; B2: tumour tissue after neoadjuvant chemoradiotherapy;

Normalised *VEGFR-1/-2 *expression of patients with LARC was significantly higher in pre-treatment tumour tissue in comparison to the corresponding normal mucosa (*VEGFR-1*; p = 0.0023 and *VEGFR-2; *p = 0.0005) but failed to be statistically significant for *TKTL1 *(p = 0.082). Accordingly, after completion of chemoradiotherapy higher *VEGFR-1/-2 *expression levels (p = 0.025; p = 0.063) were observed in tumour tissue as compared to healthy mucosa at time of surgery, whereas no differences in gene expression levels of *VEGFR-1 *and *-2 *were observed in tumour samples before and after neoadjuvant radiotherapy (Figure 1).

### Expression levels and pathological tumour response (TRG, pCR)

A total of 11 out of 33 patients showed a poor response (33%; defined as TRG of 0 or 1), while 22 patients had good response (67%; TRG 2 or 3, Table 1). Pathological T-downstaging was accomplished in 15 patients (45%, "T-responder"), 3 of whom (9%) achieved a pathological complete remission (ypT0 N0). No significant correlation between the two scores could be seen in our cohort (CI: 0,7-2,1, p-value:0,4).

Median pre-treatment expression levels of the three genes were used as a cut off value, dividing patients into low and high-expression groups (Figure 1) No significant correlation was observed neither for the JSCCR grading (TRG) nor the complete pathological response (pCR) nor for gene expression levels (Table 2).

**Table 2:** Prognostic value of pathological tumour response for *VEGFR-1*, *VEGFR-2*, and *TKTL1*

	VEGFR-1/GUS ≤ Median (n = 13)	VEGFR-1/GUS > Median (n = 13)	p-value
TRG^┼ ^2 - 3	9 (69.2%)	8 (61.5%)	p = 1.0

pCR* (ypT0 N0)	2 (15.4%)	1 (7.7%)	p = 1.0

	**VEGFR-2/GUS ≤** **Median (n = 16)**	**VEGFR-2/GUS >** **Median (n = 16)**	

TRG^┼ ^2 - 3	12 (75%)	9 (56%)	p = 0.19

PCR* (ypT0 N0)	2 (16.7%)	1 (6.3%)	p = 1.0

	**TKTL1/GUS ≤** **Median (n = 17)**	**TKTL1/GUS >** **Median (n = 16)**	

TRG┼ 2 - 3	11 (64.5%)	11 (68.8%)	p = 1.0

PCR* (ypT0 N0)	1 (5.9%)	2 (12.5%)	p = 0.6

pathological complete remission, ^┼^Tumour regression grading

### Gene Expression levels and DFS

All but one patient underwent curative surgery. The latter had an irresectable T4-tumour and revealed tumour progression with peritoneal spread during chemoradiotherapy. Median follow-up time was 33 months (range: 9-51). Local recurrence and distant metastases were recorded in two (12.5%) and 10 patients (65%), respectively.

**Figure 2:**
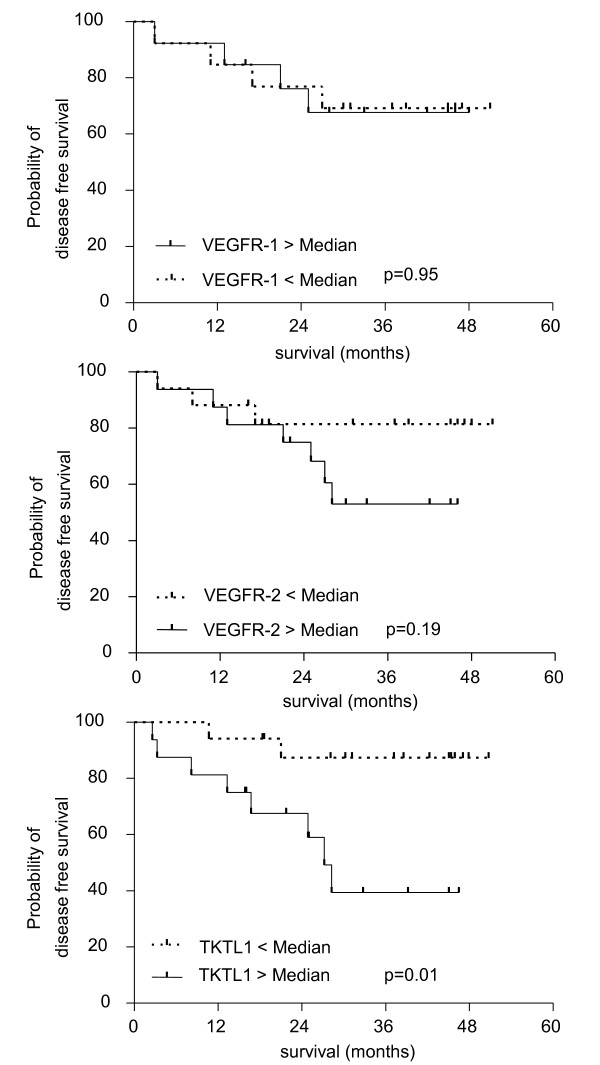
**Probability of disease-free survival correlated to gene expression levels of *TKTL1*, *VEGFR-1 *and *VEGFR-2;***. Probability of disease-free survival correlated to gene expression levels of *TKTL1*, *VEGFR-1 *and *VEGFR-2; *Median pre-treatment expression level was defined as cut-off level for the respective gene expression in the investigated rectal cancer patients.

A longer three-year DFS could be observed in patients with low *TKTL1 *expression (3-year DFS: 87% versus 39%, n = 33, p = 0.01, HR: 0.19, CI: 0,05-0,6), while three-year DFS was virtually identical for patients with high vs. low *VEGFR-1 *expression (3-year DFS: 69% versus 68%, n = 26, p = 0,9, HR: 1,04, CI: 0,26-4,2). The impact of *VEGFR-2 *expression on DFS failed statistical significance but a trend was observed regarding 3-year DFS in favour of patients with lower *VEGFR-2 *expression (3-year DFS: 81% versus 53%, n = 32, p = 0.19, HR:0,43, CI: 0,13-2,15; see Figure 2).

### Correlation of TKTL1 expression with clinical and molecular findings

The high and low expressing patient groups are compared in Table 3 with respect to several clinical and molecular parameters. Median age in patients with a higher *TKTL1 *expression was younger compared to patients with low *TKTL1 *expression. No significant differences could be seen in initial tumour stage and TRG. Serum tumour markers in the patient group with higher *TKTL1 *expression were slightly higher, but this did not reach statistical significance (p-value for CEA p = 0.07, n = 16, p-value for CA 19.9, p = 0.18, n = 16). Patients with high *TKTL1 *levels eventually developed metastases or local recurrence significantly more often than patients with low *TKTL1 *levels (11 vs. 1 pts., p = 0.0002). *TKTL1 *expression was correlated to *VEGFR-1/2 *and to *PTEN*, *KRAS *and *Survivin *expression in our cohort. Expression of the latter molecular markers has previously been published by our group [28,29]. No correlation between high *TKTL1 *expression and *VEGFR-1 *or *-2 *expression was demonstrated, while a tendency towards higher survivin expression in the *TKTL1 *overexpressing group could be detected (13 vs 17 pts., p = 0.08).

**Table 3:** Clinical and laboratory findings dependent on *TKTL1 *expression status

	TKTL1/GUS ≤ Median (n = 17)	TKTL1/GUS > Median (n = 16)	P value
**Median age (n = 33)**: 61 years	63	59	p = 0.89

**Clinical tumour stage**	uN negative n = 5 (29%)	uN negative n = 5 (31%)	p = 1.0
	uN positive n = 12 (71%)	uN positive n = 11 (69%)	

**Pathological tumour stage (n = 32)**	ypT0N0 n = 1	ypT0N0 n = 2	p = 0.69
	ypT1-2N0 n = 6	ypT1-2N0 n = 6	
	ypT3-4N0 n = 6	ypT3-4N0 n = 2	
	ypTanyN+ n = 4	ypTanyN+ n = 5	

**CEA (median)**	2.1	4.25	p = 0.074

**CA 19-9**	10	23	p = 0.18

**VEGFR1 (median)**	0.4125; n = 10	0.1034; n = 16	p = 0.19

**VEGFR2 (median)**	0.4413; n = 16	0.8241; n = 16	p = 0.11

**Survivin (n = 30) (median)**	6.4; n = 17	8.5; n = 13	p = 0.081

***KRAS *mutated (n = 14/33)**	n = 6 (35%)	n = 8 (50%)	p = 0.49

***Loss of PTEN (n = 2/30)***	n = 1/16 (6%)	n = 1/14 (7%)	p = 1.0

**Local recurrence**	n = 0 (0%)	n = 2 (12.5%)	p = 0.22

**Metastasis during follow up**	n = 1 (6%)	n = 9 (56%)	p = 0.0024

**Median DFS ∞ (months)**	39	23	p = 0.017

**Death**	n = 2 (12%)	n = 4 (25%)	p = 0.39

**Median Survival (months)**	39	26	p = 0.26

∞ disease-free survival

## Discussion

Despite intensive neoadjuvant treatment regimens using chemotherapy and radiotherapy, 35-40% of patients with locally advanced rectal cancer (LARC) eventually will develop distant metastases and die of this disease. To date, no validated prognostic or predictive molecular marker in the setting of locally advanced rectal cancer is established to tailor treatment individually to the patients.

Angiogenesis is a central process in tumour progression, and enhanced glycolytic metabolism of carbohydrates even in the presence of oxygen ("aerobic glycolysis") has been demonstrated to be involved in progression and resistance in several solid tumours [3,4,30,31]. In the present study, we evaluated the potential prognostic and predictive impact of *VEGFR-1*, *VEGFR-2 *and *TKTL1 *mRNA expression levels in patients with LARC receiving intensified neoadjuvant chemoradiotherapy with capecitabine, irinotecan and cetuximab. Quantification of the examined genes was performed using qPCR and normalized against the housekeeping gene GUS.

The main finding of this study was an inferior 3-year DFS for patients with high *TKTL1 *expression as compared to those with low *TKTL1 *expression using qPCR (3 year DFS: 39% vs 87%; p = 0.017). *TKTL1*, an altered isoform of the transketolase gene, is upregulated in many human cancers [14-17,32]. TKTL1 protein renders tumour cells autonomous by means of infinite glucose consumption irrespective of oxygen supply [13,16]. Transketolase reactions in the pentose phosphate pathway (PPP) convert glucose to ribose for nucleic acid synthesis and generate NADPH, a reducing agent required for synthesis reactions in growing tumour cells. More than 85% of the nucleic acid in certain tumours derives from ribose generated in the nonoxidative part of the PPP [33].

Shin and co-workers described an upregulation of glycolytic enzymes including Transketolase in 5-FU resistant colon cancer cell lines [34], an argument for the potential involvement of *TKTL1 *in progression and therapy resistance.

*TKTL1 *expression in colon cancer was shown to be upregulated as compared to *TKT *and *TKTL2 *expression using immunohistochemistry and cell culture assays [14,17]. Significant reduction in cell growth and viability in human LoVo and HCT116 colon cancer cells treated with *TKTL1 *siRNA as compared to LoVo/HCT116 cells without RNAi treatment in a cell culture model using quantitative PCR was demonstrated by two different groups [14,17]. Langbein et al. [16] examined untreated tumour tissue of 70 colon cancer patients and showed overexpression of TKTL1 in invasive carcinomas as compared to healthy tissue and non-invasive tumours on protein level. Overexpression of the TKTL1 protein was related to lower disease specific survival. In the same study, five colon cancer samples have been analysed by quantitative PCR, also showing *TKTL1 *overexpression in invasive carcinomas on mRNA level.

Although *TKTL1 *expression has been analysed in many solid tumours, to date no such analysis has been done for rectal cancer. Most of the studies focusing on *TKTL1 *expression in solid malignancies examined *TKTL1 *expression levels via immunohistochemistry, whereas expression on cDNA level has been examined less frequently.

Altered glucose consumption may also have therapeutic consequences: It became apparent that depletion of ATP by glycolytic inhibition potently induced apoptosis in multidrug-resistant cells in vitro [33,35]. Inhibition of the ultimate step in glycolysis (the conversion of pyruvate to lactate) has been proven to be effective in vivo and in vitro in breast cancer [36]. In addition, the activation of transketolases by application of thiamine stimulates tumour growth [37]. Specific inhibition of *TKTL1 *might be a useful target in this setting in cases, where *TKTL1 *expression is upregulated. To date, many preclinical glucose inhibitors have been developed, giving rise to a possibly new substance group in the near future [38].

In our analysis, *TKTL1 *expression significantly correlates with DFS and development of metastasis, whereas no significant difference for median survival, local recurrence rates or death could be found. The reason for this controversary again might be found in the sample size: all investigations show a trend towards better outcome in low *TKTL1 *expressing patients and should therefore be analyzed again in a larger cohort.

No correlation of *TKTL1 *expression with the other investigated markers could be detected in our study. Lack of prognostic value for *survivin expression *and *KRAS/PTEN *mutation status is in line with prior findings of our group [28,29]. A slight tendency towards higher *survivin *expression in patients overexpressing *TKTL1 *can be regarded as a possible response of tumour cells to hypoxic conditions. Survivin has been shown to being secreted as anti-apoptotic protein under hypoxic conditions [39].

In contrast to our findings concerning *TKTL1*, *VEGFR-1/-2 *expression does not seem to play a role as a predictive or prognostic marker in LARC in our cohort.

Importance of *VEGF*- as well as *VEGF*-subtype- and receptor-overexpression in solid tumours has been widely discussed in the last few years. *VEGFR-1 *is said to mediate biologic activity in human cancer cells [7], *VEGFR-2 *regulates downstream molecules such as PI3K or AKT and therewith steers endothelial differentiation, DNA synthesis and proliferation [40]. However, the actual impact of the receptors on therapeutic outcome and prognosis remains controversial. André et al. [9] found increased expression of *VEGFR-1 *and one of its ligands *VEGF-A *on mRNA level in colon cancers, but could not outline a prognostic value. Bertolini and coworkers found no significant association between baseline expression of *VEGF *(measured by immunohistochemistry) and pCR, disease free survival or overall survival analysing 91 patients with LARC. An increase in VEGF expression after the neoadjuvant treatment could be observed [41].

Another study analyzed *VEGF *expression using immunohistochemistry in 81 patients with LARC receiving neoadjuvant radiotherapy [5]. No significant correlation of *VEGF*-expression with the pCR and local relapse rate was observed while disease free survival was poor due to an increased rate of distant metastases.

In contrast, Zlobec et al. [8] analyzed 104 patients and found a significant correlation of *VEGF *levels with pCR rates using immunohistochemistry. Both studies may be criticized because pre-treatment was not standardized in the investigated patient population and differences between patients receiving radiotherapy only and those undergoing chemoradiation cannot be ruled out.

Our findings underline the involvement of *VEGFR-1*/-*2 *in primary tumour growth and progression of rectal cancer due to significantly increased levels compared to normal tissue which is in line with the results reported by others [9]. *VEGFR-1*/*-2 *expression was not altered by the chemoradiotherapy regimen applied to the present patient group. *VEGFR-1/2 *expression did not correlate with tumour regression grade or tumour downsizing. Accordingly, no significant correlation between *VEGFR-1*/-*2 *expression and DFS has been noted. However, a trend can be seen towards a better prognosis for patients with low *VEGFR-2 *expression (Figure 2). *VEGFR-2 *expression should therefore be analyzed with a larger number of patients. Several studies have analyzed gene expression by immunohistochemistry, while the present analysis has focused on mRNA expression of *VEGF*-receptors, which could explain the different results.

The lack of correlation between *TKTL1 *and *VEGFR-1/2 *expression (Table 3) suggests that many different other mechanisms are involved in tumour growth and metastasis and that a change in tumour metabolism is only one of many alterations [42]. Again, it has to be mentioned that the sample size of the cohort was relatively low due to scantness of tumour tissue available, and investigation of a larger cohort size should be done in order to prove this hypothesis.

## Conclusion

High *TKTL1 *expression strongly correlated with poor outcome in patients with LARC receiving neoadjuvant intensified chemoradiotherapy in the present analysis and may therefore be regarded as a potential prognostic marker. Further validation of *TKTL1 *in larger patient cohorts using multivariate analysis appears to be warranted. It also has to be proved, whether *TKTL1 *expression as a predictive marker can be used in patient groups with other treatment regimens apart from 5FU/cetuximab/radiotherapy. Moreover, glucose metabolism as a "druggable" target in solid tumours is under current investigation and the potential links between high *TKTL1 *expression and the metastatic potential of tumours deserves further research. Expression of VEGFR-1/-2 did not correlate with disease-free survival in our study.

## Competing interests

The authors declare that they have no competing interests.

## Authors' contributions

JS, KH, PE, PS, BB carried out the molecular genetic studies. JS, RDH and PE drafted the manuscript. RDH, PE, JS participated in the design of the study and performed the statistical analysis. Study material and/or patients were provided from DG; GK, PK, SP, WKH, FW and RDH. All authors read and approved the final manuscript.

## Pre-publication history

The pre-publication history for this paper can be accessed here:

http://www.biomedcentral.com/1471-2407/11/363/prepub

## Supplementary Material

Additional file 1 **Primer sequences of *VEGFR-1/-2 *and *TKTL1***. Primer sequences of *VEGFR-1/-2 *and *TKTL1 *used in described PCR assays. Click here for file
