# Supplementary material for: Expression of Transketolase like gene 1 (TKTL1) predicts disease-free survival in patients with locally advanced rectal cancer receiving neoadjuvant chemoradiotherapy
Source: BMC Cancer. 2011 Aug 19;11:363. doi: 10.1186/1471-2407-11-363 (PMC3176245; doi:10.1186/1471-2407-11-363)
Supplement: Additional file 1 — Primer sequences of VEGFR-1/-2 and TKTL1. Primer sequences of VEGFR-1/-2 and TKTL1 used in described PCR assays. [file 1471-2407-11-363-S1.DOC]

**Additional file 1:**

**Title:** Primer sequences of *VEGFR-1/-2* and *TKTL1*

**Description:** Primer sequences of *VEGFR-1/-2* and *TKTL1* used in described PCR assays.

| **Assay** |  | **Name** | Sequence5'-3' |
| --- | --- | --- | --- |
| ***VEGFR1* quantification** | LightCycler primer | VEGFR1-F | TCTGACGGTTTCTACAAGGAGC |
| ***VEGFR1* quantification** | LightCycler primer | VEGFR1-R | agcatgatctgatagatttcaggag |
| ***VEGFR1* quantification** | LightCycler hybridisation probe | VEGFR1-FL | GAGGACTTTTGCAGTCGCCTGAGGG-  FLuorescein |
| ***VEGFR1* quantification** | LightCycler hybridisation probe | VEGFR1-LC | LCRED640-AGGCATGAGGATGAGAGCTCCTGAGTACTC |
| ***VEGFR2* quantification** | LightCycler primer | VEGFR2-F | CTCACATGGTACAAGCTTGGC |
| ***VEGFR2* quantification** | LightCycler primer | VEGFR2-R | tgccacacgctctaggactgt |
| ***VEGFR2* quantification** | LightCycler hybridisation probe | VEGFR2-LC | TGCATCCTTGCAGGACCAAGGAGACT-LCRED640 |
| ***VEGFR2* quantification** | LightCycler hybridisation probe | VEGFR2-FL | FLuorescein-TGTCTGCCTTGCTCAAGACAGGAAGACC |
| ***VEGFR2* plasmid cloning** | Insert amplification primer | VEGFR-2-11-F | WGCTGTCTCAGTGACAAACCCA |
| ***VEGFR2* plasmid cloning** | Insert amplification primer | VEGFR-2-14-R | tgccacacgctctaggactgt |
| ***VEGFR1* plasmid cloning** | Insert amplification primer | VEGFR-1-F1 | CTGGGCAAATCACTTGGAAG |
| ***VEGFR1* plasmid cloning** | Insert amplification primer | VEGFR-1-R1 | agcatgatctgatagatttcaggag |
| ***TKTL1* quantification** | LightCycler primer | TKTL1-F | GGTATCTGTTGGTGACGATGGT |
| ***TKTL1* quantification** | LightCycler primer | TKTL1 R | CCAATGCCAAGGGGATGTGC |
| ***TKTL1* quantification** | LightCycler hybridisation probe | TKTL1-FL | GATCTTCTACCCAACTGATGCCGTCTCC-FLUORESCIN |
| ***TKTL1* quantification** | LightCycler hybridisation probe | TKTL1-RL | LCRED640- GGAGCATGCTGTTGCTCTGGC-P |
| ***TKTL1* plasmid cloning** | Insert amplification primer | TKTL1-F1 | GGTATCTGTTGGTGACGATGGT |
| ***TKTL1* plasmid cloning** | Insert amplification primer | TKTL1-R1 | CATTAAACCTCTGGATGTCGCC |
